# Integration of an automated cell culture analyzer with a closed-system hollow-fiber bioreactor for online metabolite detection and cell monitoring

**DOI:** 10.14440/jbm.2025.0046

**Published:** 2025-03-04

**Authors:** Nathan Schwab, Nathan Frank, Tonya Acker, Kelly Richardson, Mindy Miller

**Affiliations:** 1 Department of Research and Development, Terumo Blood and Cell Technologies, Lakewood, Colorado 80215, United States; 2 Department of Nova BioProfile Applications, Nova Biomedical, Waltham, Massachusetts 02454, United States

**Keywords:** Hollow-fiber bioreactor, On-line sensing, Metabolic monitoring, Nova Biomedical BioProfile FLEX2, Quantum Flex

## Abstract

**Background:**

On-line sensing technologies for expanding cell cultures are becoming essential tools for understanding metabolic activity during critical stages of expansion. These tools generate data that indicate when user interventions are required, such as harvesting cells or cell products, inducing cell differentiation, or altering growth medium inputs. The platform must reliably measure the biochemical and physiochemical properties of interest in a dependable, aseptic, and non-invasive manner to benefit users.

**Objective:**

In this proof-of-concept study, we used the Nova Biomedical BioProfile FLEX2 as a platform for metabolic and cellular measurements due to its ability to detect a range of metabolically relevant compounds, measure cell counts and viability, and acquire samples automatically.

**Methods:**

Here, we demonstrated the straightforward integration of the analyzer with the Quantum Flex™ Cell Expansion System (Quantum Flex) using an available sampling port. While this approach can accommodate both suspension and adherent cell types, this study focused on suspension cells only. In addition, we developed a simple method to integrate the sampling adapter into a Quantum cell expansion set, allowing sample collection at precise intervals.

**Results:**

In both cases, the samples were acquired automatically using the analyzer’s timing function, facilitating an automated expansion process with increased data collection frequency – previously impractical with manual sampling.

**Conclusion:**

This model provides Quantum Flex users with an option for online sensing to monitor cell expansion at scheduled intervals without requiring additional user input.

## 1. Introduction

Cell expansion is a critical step in the manufacturing of cellular therapies. Applications such as immunotherapy or regenerative medicine require cells to be grown in controlled environments to maintain precise conditions. As cellular therapies become more prevalent, expanding cells within closed and automated systems is increasingly important for maximizing reproducibility and minimizing the risk of microbial contamination. One example is the Quantum Flex™ cell expansion system (Quantum Flex), which is an automated and functionally closed expansion platform for cell culture that utilizes a hollow-fiber bioreactor (HFB) capable of growing adherent and suspension cells, as reviewed by Hulme *et al*.^1^ For adherent cell culture or conditions requiring immobilized growth factors, a coating substratum can be added, allowing for unidirectional flow through the membrane.^2^ Suspension cells do not require a coating reagent; instead, they are retained within the fibers through bidirectional containment feed. Two sizes of bioreactors are available for either application: standard (2.1 m^2^, 138 mL) and small (0.2 m^2^, 14.5 mL). Each HFB-based cell expansion occurs on the inside of the hollow fibers, the intracapillary (IC) side of the bioreactor, while further supplementation of physiochemical compounds occurs outside the hollow fibers, the extracapillary (EC) side, including gas exchange and pH equilibration. In addition, the EC compartment can also augment growing cells with small molecules, such as glucose, amino acids, and electrolytes, while the IC side can introduce serum and cytokines. The IC and EC sides of the hollow fibers are physically separated by a semi-permeable polyethersulfone membrane, allowing each to have its inlet pump and circulation, thus promoting a fully customizable growth environment due to the perfusion-based nature of the fibers.

To optimize expansion, cells must be contained within the HFB. Monitoring the progress of the growing culture primarily focuses on metabolic activity, particularly glucose consumption and lactate generation, derived from at-line or off-line measurements from medium samples collected through a sample port located in the EC tubing. In suspension-cell cultures, bidirectional containment feed can be interrupted to circulate the cells through the IC-loop sampling coil, allowing for excision and removal of cells within the tubing for measurements including density, viability, and size. Historically, metabolite and cell density measurements for Quantum Flex-expanded cells have been conducted using at-line sampling methods where samples are manually pulled and analyzed on a separate benchtop analyzer. However, advancements in sensor and detector technologies now enable both tasks to be performed online and in real-time to meet growing applications demands.

**Figure 1:**
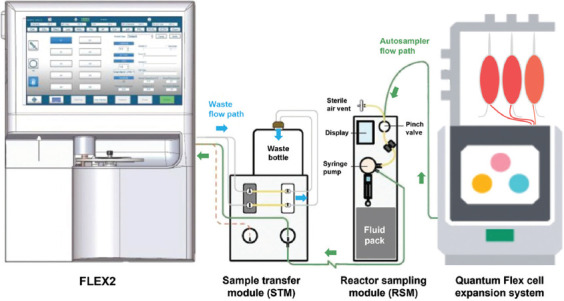
The flow path of a sample drawn from quantum flex and delivered to the FLEX2 through a RSM. A remote sampling of a Quantum Flex device using the FLEX2 is made possible by the RSM, which draws the sample out of the bioreactor through an adapter. The excess sample is transferred to the waste bottle (or a sample retainer, not shown) through the sample transfer module, while the RSM automatically cleans the line after each sample draw. Image provided by Nova Biomedical. Abbreviation: RSM: Reactor sampling module.

While spectroscopy techniques – such as Raman, mass, and near-/mid-infrared – provide a comprehensive profile of the metabolome/secretome, compared to enzyme-based methods and have steadily gained usability and application,^3^ we focused on methods that can currently integrate into the Quantum Flex platform, specifically using the Nova Biomedical BioProfile FLEX2 (FLEX2). The FLEX2 has an appropriate module (CHEMISTRY) for detecting a suite of metabolically relevant compounds, such as glucose, lactate, glutamine, ammonium, and glutamate, as well as calcium, potassium, and sodium. In addition, it includes three other modules (pH/GAS, cell density, and viability [CDV], and OSMOLARITY) for detecting physiochemical properties, such as pH, dissolved oxygen and carbon dioxide, cell density and viability, and osmolarity. To facilitate sampling directly from a bioreactor, a reactor sampling module (RSM) extracts samples and pumps them to a sample transfer module (STM), which directs the sample location (Figure 1). For analysis, the samples are moved into the FLEX2, and for retention or disposal, the samples are moved to a sample retainer (not shown) or a waste container. The RSM is programmable for scheduled sampling and/or on-demand sampling, with up to ten RSMs available for each FLEX2 analyzer.

In this proof-of-concept study, we demonstrated the integration and usability of the CHEMISTRY, pH/Gas, and CDV analytics of the FLEX2 when connected to the Quantum Flex during the expansion of primary human T cells and Jurkat cells. We found that the FLEX2 could be directly connected to the EC sample port using a currently available adapter for measuring biochemical and physiochemical compounds. It could also be modified to connect to the IC loop for cell measurements. These connections facilitated automated sampling of metabolites and cell measurements without user interventions after the initial scheduling until the harvest of the expanding culture, allowing for a completely hands-off process in the cell manufacturing workflow.

## 2. Methods

### 2.1. Cells

Jurkat cells (ATCC E6-1) were sourced from ATCC (USA), and a Quantum Flex-expanded bank was cryopreserved and maintained in the vapor phase of a liquid nitrogen storage unit.

Primary human T cells were isolated from a leukopak of a healthy donor (Cat no.: 70500, STEMCELL Technologies, Canada) using a positive CD3+ magnetic-based isolation kit (Cat no.: 17851, STEMCELL Technologies, Canada), and purity was flow cytometrically verified. These donor cells were cryopreserved and stored as described above.

### 2.2. Cell culture

Expansion of suspension cells was carried out using a Quantum Flex (Terumo Blood and Cell Technologies, USA), an updated version of the Quantum Cell Expansion System, which is a well-established HFB cell expansion system for adherent and suspension cells.^4^-^6^ Pre-determined protocols specifying feed and circulation rates for each expansion were uploaded to each Quantum Flex through the Cell Processing Application, promoting a fully automated expansion of Jurkat cells. Feed rates were manually adjusted during the culture of primary T cells due to donor variability.

Jurkat cells (ATCC E6-1) were cultured in RPMI 1640 (Fisher Scientific, USA) supplemented with 10% heat-inactivated fetal bovine serum (IC medium) or used as a base RPMI 1640 (EC medium). The expansion was performed in a Quantum Flex (Terumo Blood and Cell Technologies, USA) within a “standard” bioreactor (Cat no.: 21037, Terumo Blood and Cell Technologies, USA) until a 7-day expansion was achieved. As part of the suspension cell protocol on Quantum Flex, cells underwent a recirculation event where they were fluidically moved out of the bioreactor and through the IC tubing for 4 min before being returned to the bioreactor with fresh IC medium to resume culture. This recirculation step is critical for dissociating cell aggregation and redistributing growth nutrients. For Jurkat expansions, cells were recirculated once per day starting on day 3.

Primary human T cells were activated with anti-CD2, anti-CD3, and anti-CD28 soluble activators (Cat no.: 10970, STEMCELL Technologies, USA) for 10 min at room temperature on a nutator. Each Quantum Flex was seeded with either 3.0 × 10^7^ or 6.0 × 10^6^ T cells for the “standard” or the “small” bioreactors (Cat no.: 21037 and 21038, respectively, Terumo Blood and Cell Technologies, USA). The expansion proceeded using X-VIVO 15 (Lonza, Switzerland) with 2.0% human AB serum and 200 IU/mL of IL-2 (IC complete medium), or base X-VIVO 15 (EC medium) until the small bioreactor yielded ≥ 2.0 × 10^9^. A medium-saving strategy called “circulation feed” was employed by attaching the 5.0 L EC medium bag to both the EC line and the waste line, promoting more complete usage of the EC medium until it could no longer maintain pH above a threshold of 7.10 and lactate below 15.0 mmol/L.^7^ At this point, 5.0 L of fresh EC medium was added on days 7 and 8. Cells were recirculated through the IC loop once per day starting on day 3 of culture.

All cell expansions were performed 3 times, each on a separate device.

### 2.3. Manual sampling

Human T cell samples were manually collected during a recirculation event, a period when the expanding cells are circulated outside the bioreactor and dispersed throughout the IC loop. A sterile excision of the loop was performed using a TSCD-Q (Terumo Blood and Cell Technologies, USA) sterile tube welder. The contents of the excision were emptied into a sample cup (approximately 1.2 – 1.5 mL) and measured using the CDV module on the FLEX2 with the Load-and-Go carousel. Each cell sample measured on the FLEX2 used parameters developed in collaboration with the Nova Biomedical team to optimize measurements for Jurkat cells and primary T cells (Table 1).

**Table 1:** The value of each parameter used for cell measurements

Parameter	Value
Live cell brightness threshold	165
Live cell minimum size (diameter)	3.00 μm
Dead cell brightness threshold	80
Dead cell minimum size (diameter)	7.00 μm
Dead cell aggregate area	2500 μm^2^
Average dead cell diameter	15.00 μm
Debris size threshold	3000 μm^2^
Focus offset	−4
Settling time	90 s
Cell density multiplier	1.000

### 2.4. Automated sampling

Metabolite samples were drawn from the EC sampling port through a luer lock connection at a rate of 100 μL/s to the RSM and delivered to the STM at a rate of 50 μL/s. Sampling was limited to 3.8 mL post-prime media, using the pH/GAS and CHEMISTRY modules to analyze important metabolites, such as glucose and lactate, as well as glutamine and glutamate, potassium, sodium, ammonium, calcium, pH, and dissolved carbon dioxide and oxygen.

Automated metabolite sampling through the EC sample port was scheduled for one measurement every 6 h, starting on day 1 for Jurkat or day 3 for primary T cells, immediately before each recirculation event. All routine tasks (calibration, cleaning, data backup, etc.) were scheduled around the sampling time to avoid disruption. In addition, EC sampling was done before the daily recirculation events to allow time for system equilibration following the influx of fresh IC medium.

Automated IC sampling was performed to measure cell density, diameter, and viability through the CDV module, coinciding with the daily recirculation event. The first recirculation event was conducted manually to synchronize each device to a specific time. This time was then input into the FLEX2 Scheduling function for the respective RSM. Subsequent IC sampling was automated by the FLEX2 according to this schedule, requiring no user intervention. The daily “Feed Cells” task on the Quantum Flex was reduced from 1,440 min (24 h) to 1,433 min to accommodate the 7-min recirculation event. Each RSM was programmed to begin sampling 2 min after recirculation commenced. To prevent disruption of sample timing, post-sampling cleanup by the RSM was completed before the next sample was taken. Manual sampling from the Quantum Flex, through aseptic excision of an IC loop sample could not be performed due to the specific timing of each automated event.

## 3. Results

**Figure 2:**
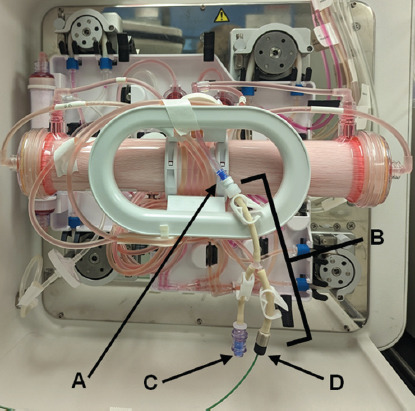
EC connection to the standard hollow-fiber bioreactor. The reactor sampling module can be connected to the EC sampling port (A) on the bioreactor through the reactor adaptor (B). This adapter allows for manual sampling or priming through a luer-lock sampling port (C) and enables the reactor sampling module to be fed through the reactor line (D). The system remains functionally closed when sampling from the EC sample port. Abbreviation: EC: Extracapillary.

Connection of the FLEX2 to a Quantum Flex HFB was performed through two intermediates: the STM and the RSM (Figure 1). This connection was facilitated by an autoclavable reactor line (Cat no.: 61370, Nova Biomedical, USA) (Component D in Figure 2) and adapter (Cat no.: 63274, Nova Biomedical, USA) that connects to a standard luer fitting (Component A in Figure 2). The luer fitting attaches to the integrated EC sampling port, which is typically used for drawing manual samples. This functionality remains intact when using the reactor line adapter (Component B in Figure 2). For suspension cell culture, the bioreactor needs to rotate during the circulation of cells throughout the IC loop, which could potentially cause the reactor line adapter to snag. We found that a single piece of laboratory tape effectively prevented this issue. In contrast, adherent cell culture involves less movement of the bioreactor and does not present any complications with this tubing adapter.

**Figure 3:**
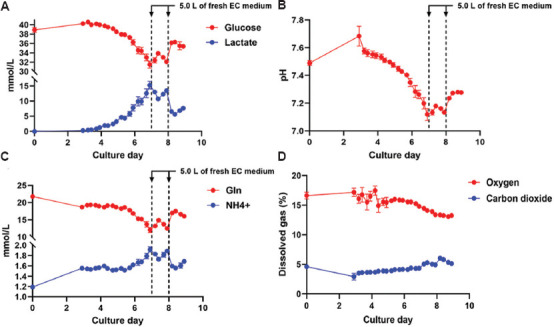
Metabolic measurements during primary T human cell expansion through EC sampling. Automated EC samples were taken every 6 h starting on day 3 to monitor the progress of the expanding culture. On days 7 and 8, 5.0 L of fresh EC medium (dashed lines) were added to keep the lactate below 15.0 mmol/L (A) and the pH above 7.0 (B). This input resulted in increased glucose, pH, and glutamine, while lactate and ammonium decreased (A, B, C, and Figure S1). In addition to measuring the decrease in glucose and increase in lactate (A), users may also observe the same relationship with glutamine and ammonium (C) or changes in oxygen and carbon dioxide (D). Each expansion and subsequent measurement was performed in triplicate. Abbreviation: EC: Extracapillary.

**Figure S1:**
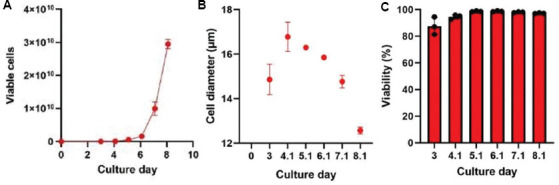
Primary T cell growth data. Cell growth and measurement data were taken off-line using the FLEX2 through the standard method of intracapillary loop excision. The cell density and viability module was used to monitor the cell density (A), diameter (B), and viability (C).

The measurement output of the FLEX2 encompasses metabolites, salts, gasses, pH, and osmolarity. During primary T cell expansion, we used lactate concentration and the resulting pH decrease to determine when to increase feed and circulation rates and assess the required input medium volume. By measuring parameters from the CHEMISTRY and pH/GAS modules every 6 h beginning on day 3, we were able to monitor the metabolic profile of the expanding culture and make informed decisions based on the generated data. After day 6 and day 7, an additional 5.0 L of fresh EC medium was connected to maintain a lactate level below 15 mmol/L (Figure 3A) and a pH above 7.1 (Figure 3B).^5^ The 6-h sampling resolution allowed for judicious augmentation of the EC medium, minimizing waste while supporting a cell culture of approximately 3.0 × 10^10^ (Figure S1). The timing of harvest was determined by cell counts, with a target expansion of at least 2.0 × 10^10^ cells. Although glutamine and oxygen levels were monitored (Figure 3C and D, respectively), they were not used to determine culture conditions during this expansion. Interestingly, an increase in glucose was seen on day 3 compared to day 0, which we hypothesize is due to residual phosphate-buffered saline remaining in the bioreactor after priming the system but before loading cells on day 0.

**Figure 4:**
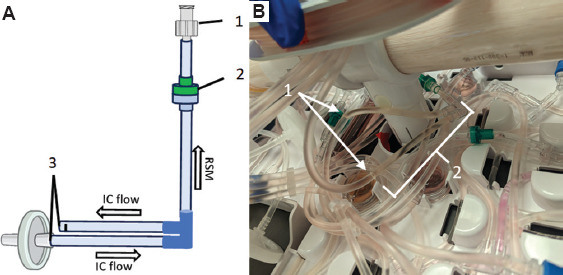
Integrating into the IC loop for cell sampling. A simple disposable was made to automate the entire cell expansion and measurement process (A). The luer fitting (A1) connected directly to the reactor line adapter for sample delivery to the reactor sampling module. A unidirectional check valve (A2) prevented the unlikely event of the sample flowing back into the IC loop. Finally, a sealed end and a sterile filter were welded off during the IC loop integration (A3), just downstream of the IC circulation pump (B). The white arrows (B1) indicate where the lines (A3) were welded into the bioreactor set, in reference to the rest of the disposable (B2). Abbreviation: IC: Intracapillary.

**Figure 5:**
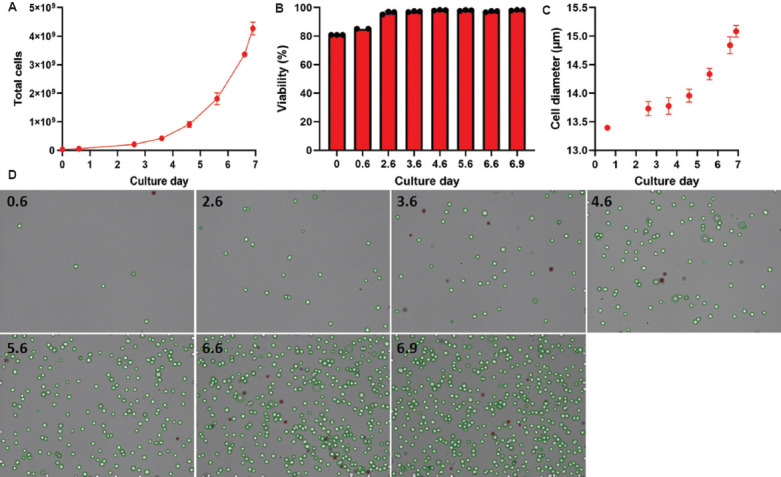
Automated cell sampling of Jurkat cells in a functionally closed cell expansion system. After aligning the timing for samples (day 0.6), all subsequent samples (every 24 h) were collected hands-free online until the final sample before harvest (day 6.9). The cell density (A), viability (B), and diameter (C) were measured using the cell density and viability module on the FLEX2, with representative images showing live (green) and dead (red) cells from the same bioreactor and flow cell position (D) on different culture days (0.6–6.9), which were used for graphic analysis. Each expansion and subsequent sampling was performed in triplicate. Note: Images are the data output from FLEX2 and do not include a scale bar.

**Figure S2:**
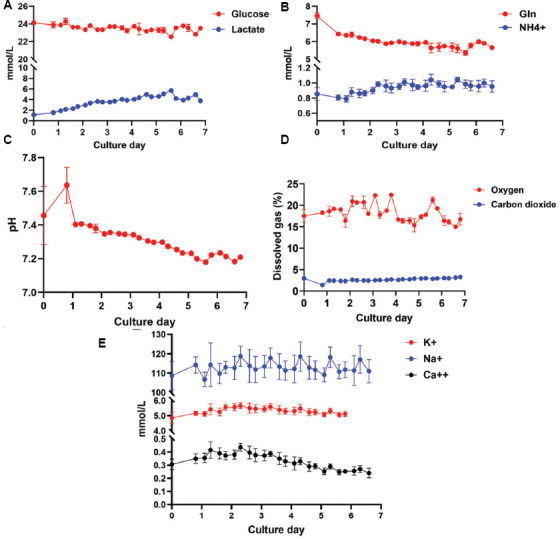
Automated metabolic and physiochemical data. Automated metabolite samples were taken through the extracapillary sample port through a secondary reactor sampling module every 6 h beginning on day 0.8 until harvest on day 6.9. The chemistry module was used to monitor relevant metabolites (A and B) for growth while the pH/GAS module was used to measure the pH and the gasses, carbon dioxide, and oxygen (C and D, respectively). Potassium, sodium, and calcium were also measured (E) but not used to influence feed or circulation rates.

The necessity to contain suspension cells within the HFB during expansion restricts the cell density measurements to occur only during recirculation events when the bidirectional flow is converted to unidirectional circulation for a period. Following this, a bolus of fresh IC medium is added through bidirectional flow to reposition cells within the fibers of the HFB. Typically, this occurs at scheduled times or manually when an operator is present to excise a piece of tubing from the IC loop during this event. In this study, recirculation events were initiated on day 3 due to a T cell lag phase characterized by minimal expansion and metabolic activity in the initial days following activation. The resulting cell counts in the loop served as a proxy for estimating total cell numbers in the bioreactor (Figure S1). To automate this process, we developed a simple disposable device that can be ethylene oxide-sterilized and welded into the IC loop (Figure 4A). We integrated this disposable immediately downstream of the IC circulation pump (Figure 4B, white arrows) to allow for free rotation of the bioreactor. The reactor line and adapter were autoclaved and assembled with the disposable in a biosafety cabinet before welding onto the bioreactor setup. The sampling time for the RSMs attached to the IC sampling lines was synchronized with daily recirculation events in the Quantum Flex protocol, enabling precise measurement of cell data. A separate RSM used for monitoring the metabolic profile of growing cells was attached to the EC sample port and collected samples every 6 h beginning on day 1 (Figure S2). This method required no user intervention for any of the three devices operating simultaneously after initial time alignment was established on day 1. Throughout the 7-day Jurkat cell expansion, the CDV module measured cell density, viability, and size (Figure 5A-C, respectively), using Trypan blue exclusion images generated by the FLEX2 (Figure 5D).

## 4. Discussion

As the number of approved cell therapies increases, there is a growing need to streamline and optimize manufacturing processes. One approach is using process analytical technologies to ensure the cellular products being manufactured meet specific quality standards throughout the process. Maintaining consistent or threshold levels of nutrients or metabolites during expansion can be beneficial for optimal cell growth. Moreover, monitoring an expanding culture is critical for generating desired functional outputs, as subtle changes can lead to physiologically relevant shifts within the cell population.^7^,^8^ Automated cell expansion devices, such as the Quantum Flex platform, require metabolite sampling to assess the health of growing cells, particularly for adherent cells that cannot be visualized inside the bioreactor. In addition, understanding the mechanical input relative to specific cellular outputs/responses within the customizable microenvironment of the HFB provides valuable feedback to the user regarding the IC and/or EC milieu. The increasing number of clinically relevant cells and cell types cultured in laboratories solidifies the need for effective monitoring to produce optimal cell products.

Manual metabolite sampling can be burdensome for technicians and introduces contamination risks during open sampling events. Many modern cell expansion platforms utilize functionally closed system sampling and automation wherever feasible. Bioanalyzer technologies such as the FLEX2, support automated sampling scheduled according to user needs. Automated sampling enables cell manufacturers to exert precise control over their end products by determining when to add nutrients, increase oxygen delivery, wash away toxic metabolic byproducts, or harvest cells. This approach can reduce waste and costs during manufacturing, as aggressive feeding cycles may not be necessary at the beginning of culture, and prolific cells may be harvested earlier compared to fully standardized expansion processes. Furthermore, automated sampling allows for an increased frequency of sampling events compared to manual processes that are limited by laboratory operating hours. For highly proliferative cell types, such as T cells, the log phase of expansion often requires multiple feed rate increases over short periods. If manual sampling occurs only once or twice a day, significant cell expansion may occur between sampling times, potentially resulting in harmful lactate accumulation. Increased sampling frequency leads to tighter environmental control and, ultimately, a better cellular product.

While the FLEX2 detects a wide range of compounds, including eight biochemical compounds, pH, pCO_2_, and pO_2_, other methods are also available. Techniques such as mass spectrometry or Raman spectroscopy can detect numerous amino acids, such as serine or arginine.^9^,^10^ which can alter the phenotype of expanding T cells.^11^,^12^ Future applications of flow cytometry or RNA sequencing could provide insights into phenotypic and transcriptomic changes during cell expansion. In addition, if a more comprehensive dataset is desired from the FLEX2, samples from each RSM draw can be retained for later analysis.

Combining metabolite detection using the FLEX2 with the Quantum Flex automated expansion system offers opportunities to enhance and fine-tune growth parameters for optimized cell expansions. This integration promotes a fully automated process for generating cells or cell products, resulting in time savings, consistency, and convenience for each sampling event. It is important to note that this work was conducted using suspension cells for measuring cell counts, as adherent cells remained attached to the bioreactor during expansion until they were proteolytically released for harvest. The utility of automated systems is evident in both early-phase researches, where constant monitoring supports process development, and late-phase manufacturing, in which established processes can be easily initiated through pre-programmed tasks with high reproducibility. Continuous collaboration between adjacent technologies ensures that cell manufacturers have access to high-quality devices that support the future of cell therapies.

## 5. Conclusion

To streamline the manufacturing of cellular therapies, we developed a proof-of-concept method for integrating an off-the-shelf bioanalyzer into a hollow fiber membrane bioreactor for automated at-line sampling of glucose, lactate, glutamine, ammonia, pH, oxygen, carbon dioxide, cell counts, and cell viability throughout suspension cell expansion.

## Data Availability

Data are available from the authors upon reasonable request.
